# Financial Self-Efficacy, Retirement Goal Clarity, and Preretirement Anxiety Among Nurses in Southeast Nigeria: The Mediating Role of Job Embeddedness

**DOI:** 10.1093/geroni/igad067

**Published:** 2023-07-13

**Authors:** Ikechukwu V N Ujoatuonu, Obinna O Ike, Lawrence O Amazue, Gabriel C Kanu

**Affiliations:** Department of Psychology, Faculty of the Social Sciences, University of Nigeria, Nsukka, Nigeria; Department of Psychology, Faculty of the Social Sciences, University of Nigeria, Nsukka, Nigeria; Department of Psychology, Faculty of the Social Sciences, University of Nigeria, Nsukka, Nigeria; Department of Psychology, Faculty of the Social Sciences, University of Nigeria, Nsukka, Nigeria

**Keywords:** Financial self-efficacy, Job embeddedness, Nurses, Preretirement anxiety, Preretirement goal clarity

## Abstract

**Background and Objectives:**

It has been evidenced that retirement transitions are accompanied by preretirement anxiety about transitioning from a work-oriented lifestyle to retirement. Most employees do not proactively address these concerns during this transitional period. Thus, identifying the factors inherent in preretirement anxiety is imperative for a positive retirement transition. This study explored the role of financial self-efficacy and preretirement goal clarity on preretirement anxiety and the mediating role of job embeddedness in such relationships among prospective retiree nurses in Sub-Saharan Africa.

**Research Design and Methods:**

This cross-sectional study used self-report measures of the Pre-retirement Anxiety Scale, Financial Self-Efficacy Scale, Retirement Goal Clarity Scale, and Job Embeddedness Scale for data collection. A total of 236 nurses participated in the study. Descriptive analyses were done to determine the bivariate correlations among the study variables, while regression-based path analysis was carried out to test the hypotheses.

**Results:**

Results revealed that goal clarity and financial self-efficacy showed a strong negative association with preretirement anxiety. Also, higher job embeddedness was negatively associated with preretirement anxiety. In addition, there was a significant indirect relationship between financial self-efficacy and preretirement anxiety, as well as preretirement goal clarity and preretirement anxiety through job embeddedness. Hence, the influence of financial self-efficacy and preretirement goal clarity on preretirement anxiety was mediated by job embeddedness.

**Discussion and Implications:**

The results emphasized that financial self-efficacy and preretirement planning are imperative for a positive perception toward retirement transition. In addition, job embeddedness should be encouraged among employees because it facilitates connectedness and interrelatedness in social fusion, ideas, and projections toward retirement transition. This connotes that the development of attachment to place and the formation of strong social ties are sacrosanct for retirement transition. These results are crucial for developing a methodology for support services for prospective employees in retirement transition.

**Translational Significance:** Evidence has shown that retirement transition is usually characterized by fear, worry, and stress due to poor planning, lack of financial self-efficacy, and job embeddedness. It is imperative for future research studies and management of health care services to take into consideration the need for financial self-efficacy, preretirement goal clarity, and job embeddedness among employees in the work environment. Thus, understanding such associations in the broader work environment and life course is apt for reducing employees’ preretirement anxiety.

## Background

Retirement from employment is a crucial life transition, and all the processes that precede it may not often be a pleasant experience for many retirees (Silver, 2023). Retirement can bring about different experiences for different people. Some may face crises such as feeling unsafe, experiencing trauma or stress, losing their job role and identity, feeling lonely while reflecting on their life's achievements or mistakes, and adapting to regrets. On the other hand, others see retirement as a time for leisure, pursuing personal interests, experiencing happiness, and being free from strenuous work activities. It is essential to recognise that retirement can be a unique and complex experience for each individual (Fisher et al., 2016). Preretirement anxiety refers to worry and apprehension that grips potential retirees to cloud their experiences and judgments when approaching retirement (Fleischmann et al., 2020). Similarly, Andel et al. (2016) defined preretirement anxiety as the fear or anxiety associated with the inability to assess gratuity, the loss of reliable income, societal power, assisting significant others in retirement, loneliness, and a lack of access to basic needs for satisfaction, well-being, and communal obligation. Ugwu et al. (2019) suggest preretirement anxiety occurs without financial preparedness, social obligation, and alienation. For nurses, a departure from work to retirement transition could present a significant nerve-racking experience of adjustment, professional role-identity detachment, and adaptation challenges (Uthaman et al., 2016). Thus, retirement transition among nurses might raise depression and preretirement anxiety (Anyebe et al., 2018).

Retirement in the nursing profession in Nigeria presents various challenges that nurses may encounter as they transition from their active years of service to retirement. Some common challenges nurses face during retirement in Nigeria include financial concerns, lack of proper retirement planning, inadequate social support, and health-related issues (Ojo & Akinwale, 2019; Oyewole & Olaolorunpo, 2019). Therefore, consistent with Ajayi and Ojo’s (2020) hypothesis, a lack of retirement planning, financial worries, and difficulties adjusting to retirement are some causes of preretirement anxiety among nurses in Nigeria.

The Pension Reform Act of 2014, which outlines the retirement age, benefits, and procedures for all workers, including nurses, governs retirement policies in Nigeria. The 60 years retirement age for nurses in Nigeria or 35 years of service, whichever comes first, is accepted for retirement. During their active years of employment, nurses must contribute only a percentage of their salary to the Contributory Pension Scheme. Benefits and allowances are not included, which the National Pension Commission manages invests in a Retirement Savings Account (RSA; Adebayo, 2016). Upon retirement, nurses are entitled to withdraw a lump sum of 25% of their total RSA balance, while the remaining 75% is paid as a monthly pension for life. However, Akintoye (2018) asserted that the nature of the pension scheme run by the Nigerian government creates room for apprehension during retirement transition as a result of delays in pension payments, mismanagement of pension funds, and corruption in the pension system, which can negatively affect nurses’ retirement transition and financial security.

Thus, the Nigerian nursing profession and its work environment face many preretirement emotions (Nnadozie et al., 2022). For instance, working as a Nigerian nurse is one of the most demanding professions as a result of a lack of retirement preparedness due to their empathetic nature toward lifesaving (Anyebe et al., 2018), increased workload with fewer incentives, and increased fatigue severity (Nnadozie et al., 2022) and decreased financial self-efficacy (Arogundade, 2016). These experiences may also be aggravated by the issues of Nigerian nurses comparing themselves with their contemporaries working in Western countries. Those in Western countries have a more robust working culture, appealing retirement benefits, organizational retirement goal assistance/support and strategies, and a well-organized framework (Anyebe et al., 2018).

Furthermore, the anchoring framework for the current study is role theory (Biddle, 1979). According to this theoretical framework, responsibilities, activities, functions, and positions of actions and behaviors set apart an employee from a distinct perspective. Thus, retirement perspectives conceive that when employees discontinue working due to retirement, social life and characteristic behavior patterns in career and retirement transition involving responsibility expand, redefine, and change across work role. Because pre-retirees roles often set out responsibilities, potentials, rules, and behaviors that individuals must encounter and accomplish to avert anxiety during retirement (Bayl-Smith & Griffin, 2014). Understanding this may be accomplished by examining life’s significant changes in roles, activities, identity, and status action (Biddle, 1986), as well as retirement and career transitions (Bordia et al., 2020). The theory helps prospective retirees envisage how best to adjust age-related retirement to change and adapt to new role issues (Bordia et al., 2020). Although, pre-retiree role losses sometimes depend on the self-rated importance of work–family–social relationship bonds and the availability of other satisfying responsibilities (Andel et al., 2016). Variation in preretirement and retirement adjustment may be attributed to the identified role, activity, and status shifts (Biddle, 1986), such as financial self-efficacy, conservation of resources and self-regulation roles, retirement goal clarity, goal setting and image roles, job embeddedness, and attachment roles. Qian et al. (2018) reported that an individual’s adjustment to retirement could only be accomplished when varying roles, responsibilities, and redefinition has been established to avert anxiety before retirement. Therefore, understanding the role demands of every individual life stage is imperative in reducing preretirement anxiety during the transition to retirement.

Generally, retirement transition correlated with financial self-efficacy and goal clarity, leading to preretirement anxiety (e.g., Asebedo et al., 2018; Ugwu et al., 2019). Showing that in financial self-efficacy, the financial resource is needed to get things done to avert depression and anxiety in preretirement. Financial self-efficacy is prospective retirees’ belief about their ability and potential in finance management to become financially self-sufficient in retirement (Lown, 2011). It involves sticking to spending plans, making progress toward financial goals, and figuring out a solution to become economically self-sufficient when faced with financial challenges. Corollary financial self-efficacy involves aptitude related to proper monetary comprehension for the better exhibition of economic behavior to accomplish fiscal comfort in preretirement and retirement (Hearty & McCarthy, 2015). Lack of financial self-efficacy could weaken psychological functioning, influence adjustment and adaptation, and possibly increase pre-retirees’ anxiety (Asebedo et al., 2018).

In line with the above assertion, the conservation of resources theory (Hobfoll, 1989) posits that employees strive to acquire, protect, and maintain tangible and intangible resources. Suggesting that employees are motivated to prevent resource loss, leading to less vulnerability to experiencing preretirement anxiety when they perceive financial self-efficacy. Thus, the idea is that financial resources are an essential aspect of an individual’s overall resource pool. Financial self-efficacy can influence how people manage and cope with financial stressors (Hobfoll & Shirom, 2000). Equally, Bandura’s (1977) social cognitive theory gives credence to this analogy. The theory posits that employees’ belief in their ability to manage their financial resources effectively reduces adverse outcomes such as psychological distress and anxiety. Thus, the perception of competence in handling financial matters, making financial decisions, and achieving financial goals is a recipe for reduced anxiety in the retirement transition.

However, when financial resources are not actualized, it is considered a stressful life situation that affects retirement goals, planning, and adjustment (Topa & Alcover, 2015). Conversely, prospective retirees with high financial self-efficacy tend to plan well to successfully handle the challenges of retirement transition (Asebedoa & Seay, 2018).

Given the possible relationship between financial self-efficacy and preretirement anxiety, Topa et al. (2018) suggested that nonactualized clear retirement goals might have a potential relationship with preretirement anxiety. For example, in Nigeria, nurses with unclear retirement goals and those who did not actualize their personal-work goals could be worried and anxious before retirement (Arogundade, 2016). Preretirement goal clarity refers to the process of gaining information, preparation practices with specific comprehensible visualization, and forecasts laid down to achieve particular objectives for general well-being prior to retirement (Stawski et al., 2003). Thus, this is with the aim to minimize possible errors that might alter the actualization of retirement ambition for better well-being and flourishing in retirement (Stawski et al., 2007). Leveraging on goal-setting theory (Locke, 1968) which emphasizes that setting clear and specific goals motivates and enhances an individual’s performance. More so, when individuals are more committed, they are likely to achieve their goals when such goals are specific, challenging, and achievable (Locke & Latham, 1990). Thus, prospective employees should have clear and well-defined goals for their retirement years, including a sense of purpose, direction, and clarity about what they want to achieve or pursue after retiring from the workforce. Ensuring a smooth retirement transition, adjustment, and overall well-being is crucial in reducing pre-retirement anxiety. It serves as a protective factor. Researchers (e.g., Abd-Elrhaman et al., 2020; Marasi et al., 2016; McEvoy & Henderson, 2012) have observed that some factors may act as a pathway in the association between preretirement goal clarity and financial self-efficacy on preretirement anxiety. Thus, job embeddedness has been identified as a potential factor (Abd-Elrhaman et al., 2020; McEvoy & Henderson, 2012). Furthermore, its integrating facets are pertinent in optimizing employees’ job performance, satisfaction, commitment, and retirement (Abd-Elrhaman et al., 2020; Karatape & Avci, 2019). However, to actualize preretirement goal clarity, nurses practice desirable behaviors such as integrating the facets of job embeddedness (fit, link, and sacrifice) on colleagues and individuals within the living neighborhood or community and significant others whom they visualize could help them arrive at their desired retirement goals (Karatepe & Avci, 2019). In addition, Mitchell et al. (2001) identified that job embeddedness could be conceptualized as a composite (facets) or single construct in identifying work outcomes. Thus, the present study conceived it as a single construct, although the facets were well-elaborated.

Job embeddedness implies the extent to which employees are enmeshed, connected, attached, or tied to their job and community (Crossley et al., 2007). Job embeddedness has twofold: organization and community components with three facets each (Mitchell et al., 2001), including fit, link, and sacrifice. Though, both “organization” and “community” are abstractions that are socially constructed to capture domains where people are potentially embedded. These facets of job embeddedness (fit, link, and sacrifice) connect nurses to their organization and community, determining how much they relate to their colleagues and individuals in that community and how to disconnect among such colleagues, organizations, and community members can cause anxiety during retirement (Abd-Elrhaman et al., 2020). “Organizational fit” implies the degree of similarity or compatibility between the employee and the organizational culture (i.e., the overlap between the individual abilities and organizational demand and a match between the individual’s interest and organizational rewards), while “community fit” implies the degree of match, similarity, or compatibility between employees and their communities of the work environment such as weather, amenities, the culture of the location where one resides, with political and religious climate. Thus, when an employee’s values, careers, goals, and plans for the future are with the larger corporate culture of the community and the demands of their immediate job (e.g., job knowledge, skills, and abilities), there is less likelihood of anxiety in retirement transition. However, “Misfit” with the organizational and community values (either organization or community-based) increases the propensity of preretirement anxiety. Thus, it could be contended that when there is a lack of “fit” between an employee’s values, career, goal, and plans for the future and the overall company culture and the responsibilities of their immediate employment, there is always the probability of preretirement anxiety during retirement transition. “Organizational link” refers to the actual connection, informal, and formal connections of employee(s) with other people, projects, locations, activities, and groups in their organization. In contrast, the “community link” refers to individuals’ ties in their work area, especially with friends, relatives, and community. Furthermore, this suggests that employees are social beings with formal or informal connections with other entities on the job. However, the more connected individuals are to their family, friends, environment (i.e., community) and connected to their work, teams, and professional group (i.e., organization), the more difficult it becomes for such employees to experience preretirement anxiety during retirement transition because of the array of bonds built across the board. The third component, sacrifice, refers to the degree to which an employee perceives the cost of leaving the job (organization) and work environment (community). The cost may be physical, psychological, and financial. Hence, employees with financial self-efficacy, preretirement goal clarity, and social attachment to the broader work environment will be relaxed during retirement. Corollary job embeddedness connotes the totality of forces (both inside and outside the work context) that influence job outcomes. Thus, the exacting blend of on-the-job (organizational: link, fit, and sacrifices with colleagues) and off-the-job (community; link, fit, and sacrifices) factors could make the transition to retirement advantageous due to the expected thriving synergy in the cohesion interaction, and attachment experienced by coworkers which transcend beyond retirement. In the job embeddedness model, both the individual’s relationship to the organization and the individual to the community are essential predictors of career transition. Thus, the lack of compatibility of these facets or the folds is a risk factor for anxiety in the retirement transition.

Drawing from work-role attachment theory (Bowley, 1973), which posits that individuals form strong emotional bonds with their work role, invariably influencing their attitudes, behavior, and outcomes during the transition to retirement. However, this depends on how integrated they feel into their work and the larger workplace, including their social connections within their organization and community. Furthermore, this aligns with Lewin’s (1951) field theory that the interaction between the individual and the broader work environment determines employees’ behavior. Invariably, a proper synergy between the individual and broader work environment (e.g., work environment, organization, community) restrain forces such as fear of retirement and uncertainty about postretirement plans. Thus, employees with broader embeddedness experience less preretirement anxiety during the retirement transition because of arrays of bonds built across the board, which can be from on-the-job or off-the-job embeddedness.

Extant studies (e.g., Holtom & O’Neill, 2004; Mitchell et al., 2001; Topa & Alcover, 2015) have shown that pre-retirees with a high level of job embeddedness, such as greater involvement in their organization, family, neighborhood, and community, are better able to cope with both inside and outside work context as a result of their work-role attachments and identifications with the significant figures in the work and community domain.

Previous studies (e.g., Abd-Elrhaman et al., 2020; Karatepe & Avci, 2019; Reitz & Anderson, 2010) have only focused on job embeddedness, extra-role behavior, self-efficacy, satisfaction, social support, age, and gender, but none has looked at the mediating role of job embeddedness on financial self-efficacy and preretirement goal clarity on preretirement anxiety among nurses. In addition, such an extended model of job embeddedness is imperative in exploring the mechanism and boundary conditions relative to financial self-efficacy and preretirement goal clarity in employees’ preretirement anxiety. Furthermore, this will give a clearer understanding of the endogenous and exogenous factors inherent in such preretirement anxiety from both the objective and subjective appraisal of inadequate role transition, which perhaps might guide the design and development of effectual support services on the part of public health institutions, management, and policymakers, thus minimizing preretirement effects on nurses. Thus, what is less clear presently is how job embeddedness may serve as a pathway through which financial self-efficacy and preretirement goal clarity influence employees’ anxiety. Therefore, our study aimed to bridge that gap by exploring the relationship between financial self-efficacy and preretirement goal clarity on preretirement anxiety among nurses and to examine the mediating role of job embeddedness in such relationships.

**Figure 1.**
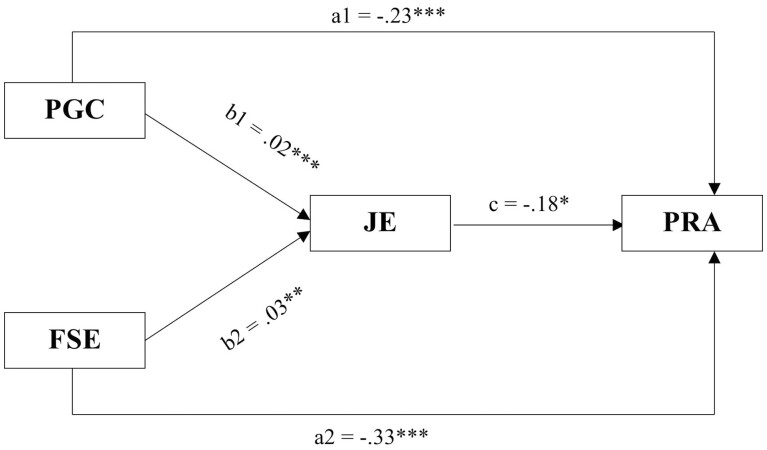
Conceptual model of the study variables. FSE = financial self-efficacy; JE = job embeddedness; PGC = preretirement goal clarity; PRA = preretirement anxiety.

The above Figure 1 gives a summative presentation of the expected patterns of association between the study variables. Relationships are expected between preretirement goal clarity and preretirement anxiety, financial self-efficacy and preretirement anxiety, job embeddedness and preretirement anxiety, the path from preretirement goal clarity through job embeddedness to preretirement anxiety, and the path from financial self-efficacy through job embeddedness to preretirement anxiety.

More specifically, the current study aimed to examine (1) the direct effect of financial self-efficacy on preretirement anxiety among nurses, (2) the direct effect of preretirement goal clarity on preretirement anxiety among nurses, (3) the direct effect of job embeddedness on preretirement anxiety, (4) mediating role of job embeddedness in the relationship between financial self-efficacy and preretirement anxiety, and (5) mediating role of job embeddedness in the relationship between preretirement goal clarity and preretirement anxiety.

Using past studies as a guide, we propose the following hypotheses: (1) Higher level of financial self-efficacy will predict lower level of preretirement anxiety among Nigerian nurses; (2) Higher level of retirement goal clarity will predict lower level of preretirement anxiety among Nigerian nurses; (3) Higher job embeddedness will significantly predict lower level of preretirement anxiety among Nigerian nurses; (4) Job embeddedness will mediate the relationship between financial self-efficacy and preretirement anxiety among Nigerian nurses; and (5) Job embeddedness will mediate the relationship between preretirement goal clarity and preretirement anxiety among Nigerian nurses.

## Method

### Study Design

The current study is a quantitative cross-sectional survey conducted with nurses from government-owned federal hospitals in Southeast Nigeria between September 2021 and February 2022. Government-owned federal hospitals represent large general hospitals in Nigeria, with the highest health care provider in human resources, medical technology, quality health care, and patient safety (Nnadozie et al., 2022). Furthermore, this is pertinent because these hospitals hire qualified nurses to absorb them until retirement. Thus, their perception of preretirement goal clarity, financial self-efficacy, and job embeddedness reflects the study hypotheses.

### Participants

Purposive sampling (Palinkas et al., 2013) was employed to recruit nurses from government-owned federal hospitals in Southeast Nigeria. A total of 236 nurse participants participated in the study. The participants’ ages ranged from 50 to 60 years (mean age = 44.65; standard deviation = 4.99). The sample size was calculated with a 5% margin error, a 95% confidence interval (CI), and an estimated sampling population of 537 enlisted as nurses between 50–60 years or who have 10 years to their retirement service years; the recommended minimum sample size using the Raosoft online sample calculator (https://www.raosoft.com/samplesize.html (accessed on September 2021) was 225 participants. Thus, the sample size utilized in the study was above the threshold level. The inclusion criteria for the study were male and female nurses aged 50–60 years or less than 10 years before retirement or years of service from (25–35 years). We sampled these participants because they have characteristics that are of interest to the study. However, 10 years or less before retirement could be a time that those with impending retirement are more likely to start brooding about the implication of being a retiree, as reported by these studies (e.g., Topa et al., 2018; Ugwu et al., 2019). For the demographic data of the study participants, see Table 1.

**Table 1.** Participant Characteristics (*N* = 236)

Demographic	Frequency	Mean (*SD*)	%
Gender			
Male	60		25.4
Female	176		74.6
Age (range 50–60 years)		44.65 (4.99)	
Educational qualification			
BSc Nursing/Registered Nurse	196		83.1
MSc Nursing	40		16.9
Marital status			
Single	20		8.5
Married	204		86.4
Divorced/widowed	12		5.1
Length of service			
25–29 years	187		79.2
30–above	49		20.8
Number of dependents			
1–4	73		30.9
5–10	137		58.1
11–above	26		11.0

*Note*: *SD* = standard deviation.

### Measures

The questionnaire consists of two parts; demographic data such as gender, age, marital status, year of service, and educational qualification were elicited from the participants before completing the self-report measures of preretirement goal clarity, financial self-efficacy, job embeddedness, and preretirement anxiety.

### Nigerian Pre-retirement Anxiety Scale

Ugwu et al. (2019) developed the 15-item Nigerian Pre-retirement Anxiety Scale to measure preretirement anxiety. The instrument is designed to ascertain employees’ worries and apprehensions when approaching retirement based on financial preparedness, social obligation, and social alienation. The scale is rated on a 5-point Likert scale that ranges from 1 = Strongly Disagree to 5 = Strongly Agree. Sample items include: “I will not want to retire because I will not be able to offset my health-related hospital bills.” “I am afraid that I will be lonely when I retire.” “Retirement makes me lose societal power at home and in my community.” Higher scores indicate more significant preretirement anxiety. Ugwu et al. (2019) reported a Cronbach’s alpha reliabilities coefficient of 0.83 for Financial Preparedness, 0.75 for Social Obligation, and 0.70 for Social Alienation, with an overall score of 0.73. Ugwu et al. (2019) asserted that it could be used as a composite or single construct. The current study reported a Cronbach’s alpha of 0.79 for Financial Preparedness, 0.72 for Social Obligation, and 0.79 for Social Alienation, with an overall score of 0.82. In this present study, it was used as a single construct.

### Financial Self-Efficacy Scale

Financial Self-Efficacy Scale (FSES), developed by Lown (2011), was used to assess the extent of nurses’ confidence and challenges before retirement. The FSES is a six-item scale, scored on a 4-point Likert response format that ranges from 1 = not true at all to 4 = exactly true. Sample items include; “I worry about running out of money in retirement.” “When faced with a financial challenge, I have a hard time figuring out the solution.” “It is hard to stick to spending plans when unexpected expenses arise.” Higher scores indicate greater financial self-efficacy. Lown (2011) reported a Cronbach’s alpha reliability of 0.76. The present study yielded acceptable internal consistency reliability of 0.77.

### Retirement Goal Clarity Scale

The Retirement Goal Clarity Scale (Stawski et al., 2007) assessed employees’ comprehensible ambition for retirement, expectations regarding their quality of life, how much they needed to save, and plans with a spouse, friends, and significant others for retirement. It is a five-item scale measured on a 7-point Likert response format ranging from 1 = Strongly Disagree to 7 = Strongly Agree. Sample items from the scale include; “I have thought a great deal about the quality of life in retirement.” “I have a clear vision of how life will be in retirement.” Stawski et al. (2007) reported a Cronbach’s alpha of 0.90. A higher score indicates greater retirement goal clarity. The present study yielded a Cronbach’s alpha of 0.86.

### Job Embeddedness Scale

Job Embeddedness Scale (Crossley et al., 2007) was used to measure nurses’ on-the-job (work) and off-the-job (community) job embeddedness. It is a 12-item scale, measured on a 5-point Likert scale response format ranging from 1 = Strongly Disagree to 5 = Strongly Agree. Sample scale items include; “The organization provides me with a way of life that suits me.” “Even if I decide to leave the organization, I would still live in the area where I am based at the moment.” “I would be very sad to leave the general community where I am based right now.” Crossley et al. (2007) reported a Cronbach’s alpha coefficient of 0.91. Higher score indicates greater job embeddedness. The present study reported a Cronbach’s alpha coefficient of 0.89, comparable to Crossley et al. (2007).

### Data Collection

Data for the current study came from 236 nurses from four government-owned federal public hospitals in Southeast Nigeria. After approval by the Ethical Committee of Department of Psychology, University of Nigeria, Nsukka (Approval No: D.PSY.UNN/REC/2022-1-1RB00010), the researchers sought the permission of the various hospital management used for the study, which they obliged. We informed the participants of the purpose of the study and eligibility criteria. The participants consented to the study by ticking √ the consent box at the questionnaire’s top. No means of identification were included in the questionnaire to promote respondents’ confidentiality. Data were collected through self-report measures between September 2021 and February 2022 through purposive sampling. We distributed copies of the questionnaire to them in their wards and units. The questionnaire was completed between 15–20 min, and the filled copies were retrieved for approximately 2 months. Out of the 243 copies of the questionnaire distributed, seven were not correctly filled and so were discarded. The collected copies of questionnaire forms used were 236 showing a return rate of 87.4%, which is acceptable.

### Statistical Analyses

A prelusive analysis was conducted to ascertain the means, the standard deviations, and the bivariate correlations among the study variables using SPSS version 21 (IBM Corp., Armonk, NY). Then, regression-based path analysis was carried out based on 2,000 bias-corrected bootstrapped samples to test the hypotheses as done in PROCESS for SPSS macro version 2.13.2 (Hayes, 2018). Bootstrapping help to statistically and empirically gather adequate samples from the original sample in mediation analysis. The idea is based on the reality that the initial sample is a small representation of the population, necessitating a random replacement sample taken from the original sample to obtain a larger sample size. In addition, a case may be included more than once in a bootstrapped sample to resemble the studied population (Hayes, 2018). Mediation exists if the bias-corrected bootstrap CI is statistically different from zero (Abu-Bader, 2021). The bias-corrected bootstrap CI adjusts for any bias in the end points of bootstrap estimates (Hayes, 2018). The explanatory, mediator, and response variables are all inputted simultaneously while performing regression-based path analysis with PROCESS, and the results of the path coefficients connecting the variables are displayed. Because they were not entered at distinct steps, this method does not rely on the mediator’s entry lowering the coefficient between the predictor and criterion variables. This strategy has been effectively used in previous research (such as by Nunfam et al., 2022) and is compatible with the recommendations (Hayes, 2018).

### Ethical Considerations

Ethical approval (blinded for review). All procedures followed were under the ethical standards of the responsible committee on human experimentation (institutional and national) and the Helsinki Declaration of 1975, as revised in 2000.

## Results

Regarding sample size, 176 (74.6%) were female nurses, while 60 (25.4%) were male nurses. For marital status, 204 were married, representing 86.4%, with 12 single nurses representing 5.1% and 20 widowed nurses representing 8.5%. Finally, for educational qualification, 196 had either a Bachelor of Science certificate or were Registered Nurses representing 83%, and 40 had a Master of Science, representing 17% (see Table 1).

Table 2 showed that age did not correlate with any of the variables in the study but correlated with length of service (*r* = 0.58, *p* < .001) and retirement age (*r* = 0.38, *p* < .01). Gender correlated with job embeddedness (*r* = −0.25, *p* < .001), length of service was negatively correlated with retirement age (*r* = 0.26, *p* < .001), but was positively correlated with preretirement anxiety (*r* = 0.31, *p* < .001). Retirement age had a negative correlation with retirement goal clarity (*r* = −0.17, *p* < .01). Number of dependents had a negative relationship with financial self-efficacy (*r* = −0.16, *p* < .05), while educational qualification correlated negatively with retirement goal clarity (*r* = −0.14, *p* < .05). Financial self-efficacy was positively correlated with retirement goal clarity (*r* = 0.32, *p* < .001), and negatively correlated with preretirement anxiety (*r* = −0.41, *p* < .001). Retirement goal clarity negatively correlated with preretirement anxiety (*r* = −0.16, *p* < .05), while the relationship between job embeddedness and preretirement anxiety was positively correlated (*r* = 0.25, *p* < .001).

**Table 2.** Pearson’s Correlations of Demographic Characteristics, Financial Self-Efficacy, Retirement Goal Clarity, Job Embeddedness, and Preretirement Anxiety

Variable		Mean	*SD*	1	2	3	4	5	6	7	8	9	10
1	Age	44.65	4.99	—									
2	Gender	—	—	0.04	—								
3	Length of service	25.61	4.87	0.58***	−0.06	—							
4	Retirement age	60.83	3.01	0.38**	0.11	−0.26***	—						
5	Number of dependents	5.60	1.78	0.09	−0.02	0.01	0.06	—					
6	Education	—	—	−0.06	−0.05	−0.00	0.07	0.09	0.12	—			
7	Financial security	14.47	4.36	−0.06	−0.07	−0.01	−0.08	−0.16*	0.09	−0.04	—		
8	Retirement goal clarity	25.22	7.89	−0.10	−0.10	0.01	−0.17**	−0.11	−0.14*	−0.01	0.32***	—	
9	Job embeddedness	36.11	8.64	0.19	−0.25***	0.09	0.10	0.10	0.01	0.12	0.06	0.02	—
10	Preretirement anxiety	43.70	8.93	0.11	−0.01	0.31***	−0.03	0.01	0.08	−0.08	−0.41***	−0.16*	0.25***

Notes: *SD* = standard deviation. Gender (0 = female, 1 = male); educational status (0 = Registered Nurse/Bachelor’s degree, 1 = Masters’ degree).

***p < .001. **p < .01. *p ≤ .05.

### Direct and Indirect Effects

The results in Table 3 from regression-based path analysis show that preretirement goal clarity (estimate = −0.23; 95% CI [−0.16, −0.29]; *p* < .001) and financial self-efficacy (estimate = −0.33; 95% CI [−0.25, −0.41]; *p* < .001) had a direct negative and significant relationship with preretirement anxiety which supports H1 and H2, respectively. Job embeddedness had a direct negative and significant relationship with preretirement anxiety (estimate = −0.18; 95% CI [−0.11, −0.27]; *p* = .048), which supports H3.

**Table 3.** Regression-Based Path Analysis

Pathway	*B*	*SE*	BC 95% CI		*p*
			Lower	Upper	
Direct effect					
PGC → PRA	−0.23	0.42	−0.16	−0.29	<.001
FSE → PRA	−0.33	0.18	−0.25	−0.41	<.001
JE → PRA	−0.18	0.13	−0.11	−0.27	<.048
Indirect effect					
PGC → JE → PRA	0.02	0.07	0.01	0.04	<.001
FSE → JE → PRA	0.03	0.18	0.01	0.07	<.01

*Notes*: BC = bias-corrected; CI = confidence interval; FSE = financial self-efficacy; JE = job embeddedness; PGC = preretirement goal clarity; PRA = preretirement anxiety; *SE* = standard error. BC bootstrapping results were based on 2,000 bootstrapped samples. Direct and indirect pathways from FSE and PGC to JE to PRA.

There was a significant indirect relationship between financial self-efficacy (estimate = 0.03; 95% CI [0.01, 0.07]; *p* < .01) and preretirement anxiety as well as preretirement goal clarity and preretirement anxiety through job embeddedness (estimate = 0.02, 95% CI [0.01, 0.04]; *p* < .001). Hence, the influence of financial self-efficacy and preretirement goal clarity on preretirement anxiety was mediated by job embeddedness, thereby supporting H4 and H5.

## Discussion

The present study focuses on the mediating role of job embeddedness in the associations of financial self-efficacy and retirement goal clarity on preretirement anxiety among nurses. First, it was hypothesized that financial self-efficacy would negatively affect preretirement anxiety among nurses. The current study result revealed that financial self-efficacy was negatively associated with the preretirement anxiety of nurses, thus indicating that increased financial self-efficacy prior to retirement leads to the experience of less preretirement anxiety among nurses. This study finding is consistent with previous studies (e.g., Arogundade 2016; Asebedoa & Seay, 2018; Kadoya & Khan, 2017; Nolan & Doorley, 2019; Tang et al., 2019), which found that financial self-efficacy and outcomes have significant relationships with preretirement and retirement anxiety. Drawing from the conservation of resource theory (Hobfoll, 1989), pre-retirees are instinctively aggravated to defend, hold on to, and recover these valued resources accrued prior to retirement to avert preretirement anxiety. Finally, this is pertinent because an individual’s valued material and financial resources create less stress, worry, and apprehension after retirement.

Another central finding in the study is that retirement goal clarity has a significant negative association with preretirement anxiety. Our finding showed that increased retirement goal clarity was associated with reduced preretirement anxiety. The result aligns with previous findings, which showed a negative association between employees’ goals, retirement expectations, and retirement anxiety (e.g., Uthaman et al., 2016; Zhu & Chou, 2018). Furthermore, the finding demonstrates that setting goals before retirement and actualizing such expectations and financial needs reduces preretirement anxiety. Furthermore, this is evident in goal-setting theory (Locke & Latham, 1990), which states that goals are internal representations of desired states that steer individuals’ actions and events and motivate specific behavioral patterns (e.g., being happy, relaxed, volunteering, and spending time with family). Thus, having a clear blueprint for retirement goals and actualizing such expectations is an important aspect linked to various outcomes such as satisfaction, fulfillment, and planning; thus, a recipe for reduced preretirement anxiety. Hence, estimating retirement savings needs is essential in planning activities ahead of retirement (Bi et al., 2017). In addition, Brougham and Walsh (2005) asserted that preretirement planning involves directed and goal-oriented behaviors, in which employees dedicate efforts to have a better retirement life, and that such behavior allows pre-retirees to build up reasonable anticipation of the experience of alteration during the retirement transition and lay down clear long-term goals as resources for postretirement life (Brougham & Walsh, 2007).

As expected, job embeddedness is associated with nurses’ preretirement anxiety, and the result showed that increased job embeddedness is associated with reduced preretirement anxiety among nurses. The finding aligns with previous studies, which evidenced the link between job embeddedness and preretirement anxiety (e.g., Abd-Elrhaman et al., 2020). However, perceived high job embeddedness might cushion preretirement anxiety among nurses because of the array of bonds built across the board. Studies have found evidence of the link between job embeddedness and related factors (e.g., Abd-Elrhaman et al., 2020; Karatepe, 2012; Karatepe & Avci, 2019; McEvoy & Henderson, 2012; Reitz & Anderson, 2010). These related factors such as career plateau, self-efficacy, employee outcomes (e.g., commitment, job satisfaction, and intention to leave and behavioral outcomes such as effort, motivation, cooperation, and organizational citizenship), perceived organizational support, retention of workers nearing retirement, and community attachment had shown a significant positive relationship with job embeddedness.

The result aligns with the work-role attachment theory by Bowlby (1973, 1979), which states that psychological features attach well with known ties among employees for better well-being, interpersonal, and quality of life. Some employees may have strong attachments with other employees and significant others (e.g., leaders, mentors, supervisors, colleagues, significant others within the family, social life, and living in a neighborhood; Yip et al., 2017). Bowlby (1973) further asserts that psychological attachment behaviors are adaptive and normal reactions to key attachment figures (i.e., individuals who provide support, protection, and care). As a result, the more nurses have a close attachment with significant figures in the off-the-job context, the more such nurses become less anxious and apprehensive about retirement.

Furthermore, job embeddedness mediated the association between financial self-efficacy and nurses’ preretirement anxiety. The present study result supports findings from past literature that found job embeddedness as a pathway in the relationships between retirement and retirement anxiety (Abd-Elrhaman et al., 2020; Karatepe, 2012; Karatepe & Avci, 2019). Invariably, cohesion in the broader work environment ushers in job embeddedness facets (link, fit, and sacrifice) that connect employees, such as investment making (cooperatives) and estimation of financial needs, which helps ameliorate preretirement anxiety. Furthermore, this is evident in Lewin’s (1951) field theory, which postulated that people have a perceptual life space in which the aspects of their lives are represented and connected with people and places significant to them. Equally, such people or place help them estimate and plan their financial needs and estimation. Thus, a major prerequisite for a successful transition to the retirement community is job embeddedness, which connotes the development of attachment to place and the formation of strong social ties (Mitchell et al., 2001). In addition, proper needs estimation and management of income and expenditure during the service years lead to prudent spending and investment (financial self-efficacy) that cushions the transition year of retirement, thus reduces retires preretirement anxiety (Gunaratne & Nov 2015).

This result aligns with Bandura’s social cognitive theory of self-regulation (1977), which states that self-efficacy is a mental construct that links beliefs and abilities to accomplish the route of acts needed to create performance and goal attainment. The social cognitive theory of self-regulation has a principal influence on prospective retirees thinking, drive, actions, and performance. Thus, the regulation of the pre-retirees financial self-efficacy beliefs is orchestrated by their fit and links with colleagues and significant others before and after their retirement, which will determine the preretirement transition of such retirees.

In addition, job embeddedness mediated the association between retirement goal clarity and nurses’ preretirement anxiety. Even though there is a dearth of studies on the mediating role of job embeddedness on such relationships, extant studies on retirement goal clarity (e.g., Stawski et al., 2007; Zhu & Chou, 2018) affirm that preretirement goal clarity significantly influences retirement transition. However, attachment, interaction, and exchanging ideas and views bind employees in discussing and projecting goal clarity before retirement, subject to how much employees are enmeshed in their job. As such, these multiple attachments and strong social ties among employees give room for knowledge sharing, projection of ideas, and discussion of retirement transition among workers. Furthermore, this invariably allows them to brainstorm their lives after retirement and subsequently plan by creating and mapping out set goals during service years that will help them adjust properly during retirement.

The finding aligns with the goal-setting theory (Locke & Lathan, 1990), which explains how employees are influenced to be motivated and committed to their set measurable targets and aspirations. Thus, preretirement planning involves goal-oriented behavior employees exhibit to better their retirement lives. As a corollary, such behaviors build up reasonable anticipation for the alteration roles experienced during retirement transition and lay down clear long-term goals as resources for postretirement life. However, such retirement goal clarity is projected with anticipating links and fit with colleagues, place of the neighborhood, significant others, and organized behavior.

### Implications

The study variables, job embeddedness, financial self-efficacy, and preretirement goal clarity, have proved valuable tools in job outcomes among nurses by showing their association with preretirement anxiety. In addition, evidence has shown that financial self-efficacy and preretirement goal clarity bolster happiness and life satisfaction after retirement. Thus, the findings of this study pave the way for increased awareness and orientation on the need for workers to be proactive about savings, investments, articulated goals, expectations, and pursuits during their work lives. Furthermore, this will help such employees to be financially viable and fulfilled based on the actualization of estimated goal clarity, thereby reducing the preretirement anxiety of such employees. In addition, job embeddedness makes employees feel they are part of the management and are attached to significant others. Finally, this is achieved through developing a link and fit between work and home rather than instituting programs, practices, and regulations that exacerbate social separation.

Similarly, nursing management needs, amid their professional responsibility, to show related community embeddedness through off-work social interaction and informal get-togethers. Also, this will foster a sense of social bond and attachment among these nurses in their job and community, thus facilitating connectedness and interrelatedness in social fusion, ideas, and projections toward retirement transition. Furthermore, this will invariably reduce preretirement anxiety because there is already an existing attachment, bond, and social fusion between the employees and the broader work environment, which reduces the vulnerability of anxiety during and after retirement, even when such employees have retired.

From the theoretical perspective, this study adds to the literature on preretirement anxiety by showing the importance of the theories of social cognitive theory of self-regulation, conservation of the resource theory, goal-setting theory, and work-role attachment theory in explaining the associations of the relationships among the study variables. Thus, the researchers have contributed to the literature on preretirement anxiety among employees from a theoretical standpoint by analyzing these integrated support theories and their functions in explaining the vulnerability of employees’ anxiety during and after retirement.

Empirically, the current study’s findings have filled a gap in the body of knowledge on preretirement anxiety among nurses by being the first to explore the pathway through which job embeddedness mediates the link between financial self-efficacy, preretirement goal clarity, and preretirement anxiety. Thus, it will be a springboard for subsequent researchers to explore other variables that can act as a mechanism or boundary condition for preretirement anxiety. This study adds to the existing knowledge in retirement via financial self-efficacy, retirement goal clarity, and job embeddedness as they influence preretirement anxiety.

Furthermore, the current study advocates a paradigm shift in intervention supports for prospective retirees that will incorporate the provision of social security/social protection programs (e.g., welfare scheme), support services for investment, needs estimation and financial self-efficacy and planning prior before retirement to ameliorate the experiences of preretirement anxiety in the course of retirement and anxiety after retirement.

### Limitations

The study has some limitations. One of such limitations is the small sample size because the study is performed in only one geographical area of Nigeria, which could affect the generalization of the findings. Therefore, further studies should include other geographical regions in Nigeria. Another limitation is that job embeddedness is measured in this study as a single construct, which might affect the facet’s outcomes independently. Therefore, future research should study job embeddedness as a composite construction to independently identify the facets outcome of preretirement anxiety vis-a-vis other variables under study. Furthermore, household socioeconomic status should be explored with preretirement anxiety because it can affect an employee’s financial self-efficacy and setting of preretirement goal clarity. In addition, other variables like social support, household socioeconomic status, and career transition patterns should be considered factors prone to preretirement anxiety.

## Conclusion

Despite the restrictions mentioned earlier, the current study contributed immensely to expanding the interactive effect of job embeddedness on important organizational outcomes such as financial self-efficacy, retirement goal clarity, and preretirement anxiety. As such, the current study underscores the importance of financial self-efficacy, job embeddedness, and preretirement goal clarity among employees. Such virtues enhance nurses’ financial prudence and lead to the establishment of set goals prior to retirement in order to cushion the effect of anxiety before and after retirement. Also, the researchers advocate support services and effective intervention strategies for employees during service years. Also, this is imperative, bearing in mind that the Nigerian health care system is not undergoing any major restructuring (e.g., pension reform); thus, policymakers should consider palliatives that will aid in need estimation and financial self-efficacy. Thus, such strategies might reduce preretirement anxiety from a lack of perceived financial self-efficacy and goal clarity. Furthermore, opening to diverse work practices must be supported to enable them to build up a broad array of professional skills and experiences to develop tactics and career transition planning. In addition, future studies should consider other variables like social support and career transition patterns to improve retirement goal clarity and financial self-efficacy.

## Data Availability

The data sets generated and analyzed during the current study will be available from the corresponding author upon reasonable request.
